# Posterior thalamic nucleus axon terminals have different structure and functional impact in the motor and somatosensory vibrissal cortices

**DOI:** 10.1007/s00429-019-01862-4

**Published:** 2019-03-27

**Authors:** Diana Casas-Torremocha, César Porrero, Javier Rodriguez-Moreno, María García-Amado, Joachim H. R. Lübke, Ángel Núñez, Francisco Clascá

**Affiliations:** 1 Department of Anatomy and Graduate Program in Neuroscience, School of Medicine, Autónoma de Madrid University, Calle Arzobispo Morcillo 4, 28029 Madrid, Spain; 2 Institute of Neuroscience and Medicine INM-10, Research Centre Jülich GmbH, 52425 Jülich, Germany; 3 Department of Psychiatry, Psychotherapy and Psychosomatics, RWTH Aachen University, Aachen, Germany; 4 JARA-Brain Medicine, Aachen, Germany

**Keywords:** Cerebral cortex, Thalamocortical projections, Higher order thalamus, NMDA receptors, Metabotropic receptors, Excitatory synaptic boutons

## Abstract

Rodents extract information about nearby objects from the movement of their whiskers through dynamic computations that are carried out by a network of forebrain structures that includes the thalamus and the primary sensory (S1BF) and motor (M1wk) whisker cortices. The posterior nucleus (Po), a higher order thalamic nucleus, is a key hub of this network, receiving cortical and brainstem sensory inputs and innervating both motor and sensory whisker-related cortical areas. In a recent study in rats, we showed that Po inputs differently impact sensory processing in S1BF and M1wk. Here, in C57BL/6 mice, we measured Po synaptic bouton layer distribution and size, compared cortical unit response latencies to “in vivo” Po activation, and pharmacologically examined the glutamatergic receptor mechanisms involved. We found that, in S1BF, a large majority (56%) of Po axon varicosities are located in layer (L)5a and only 12% in L2–L4, whereas in M1wk this proportion is inverted to 18% and 55%, respectively. Light and electron microscopic measurements showed that Po synaptic boutons in M1wk layers 3–4 are significantly larger (~ 50%) than those in S1BF L5a. Electrical Po stimulation elicits different area-specific response patterns. In S1BF, responses show weak or no facilitation, and involve both ionotropic and metabotropic glutamate receptors, whereas in M1wk, unit responses exhibit facilitation to repetitive stimulation and involve ionotropic NMDA glutamate receptors. Because of the different laminar distribution of axon terminals, synaptic bouton size and receptor mechanisms, the impact of Po signals on M1wk and S1BF, although simultaneous, is likely to be markedly different.

**Electronic supplementary material:**

The online version of this article (10.1007/s00429-019-01862-4) contains supplementary material, which is available to authorized users.

## Introduction

Rodent brains compute relationships between motor commands to whisker muscles and signals from whisker mechanoreceptors to infer the position, size and texture of objects in their near environment. These computations are carried out by multi-level sensorimotor neuronal loops (reviewed by Ahissar and Oram 2015) that include the “first-order” ventral posteromedial (VPM) and the “higher order” posterior (Po) thalamic nuclei as well as the whisker domains of the motor (M1wk) and primary somatosensory (S1BF) cortices (Theyel et al. 2010; Sherman and Guillery 2013).

M1wk and S1BF cortices direct exploratory whisker movements (Matyas et al. 2010; Ahissar and Oram 2015). In both areas, pyramidal neurons in layer (L)5 innervating the brainstem face motor and sensory centers send axonal collaterals to Po, where they are able to drive thalamic neurons (Urbain and Deschênes 2007; Theyel et al. 2010; Groh et al. 2014; Economo et al. 2018). Po neurons also receive ascending sensory information from the trigeminal nuclei (Veinante and Deschênes 1999; De Chazeron et al. 2004; Guy et al. 2005) and may thus carry out dynamic computations between the motor commands and the ascending sensory signals (Groh et al. 2014). In turn, Po neurons have been shown to monosynaptically excite S1BF pyramidal neurons “in vitro” (Petreanu et al. 2009; Audette et al. 2018) and evoke spike firing “in vivo” (Gambino et al. 2014; Jouhanneau et al. 2014; Mease et al. 2016; Castejon et al. 2016). Although less well characterized, Po inputs to M1wk have been shown likewise to excite cortical neurons “in vitro” (Hooks et al. 2013) and “in vivo” (Casas-Torremocha et al. 2017).

There is growing evidence suggesting that the functional impact of Po inputs reaching M1wk and S1BF might be different. Anatomical studies in rats and mice have shown that Po axons target both deep and superficial layers in S1BF while arborizing mainly in superficial layers in M1wk (Herkenham 1986; Wimmer et al. 2010; Ohno et al. 2012; Hooks et al. 2015). Moreover, functional “in vitro” studies indicate that Po synapses in S1BF activate mainly the basal dendrites of L5a pyramidal cells and, more weakly, the apical tufts of the same cells in L1 (Petreanu et al. 2009; Audette et al. 2018), while preferentially driving L2–3 and L5a neurons in M1wk (Hooks et al. 2013). In rats, we observed marked differences in the impact of Po inputs on tactile information processing in S1BF and M1wk “in vivo”. While Po stimulation interfered with the responses in both areas, the effect was layer specific in each area. Interestingly, Po silencing drastically reduced the whisker responses in M1wk cortex but not in S1BF (Castejon et al. 2016; Casas-Torremocha et al. 2017).

It is conceivable that the above differences reflect, at least in part, variations between areas in the number of Po synapses across cortical laminae, in synapse structure and/or in specific postsynaptic transduction mechanisms. In this context, it is of note that Viaene et al. (2011a) reported that the mean size of Po axonal varicosities labeled by tracer injections in the thalamus and measured using light microscopy seems to be smaller in primary somatosensory area (S1) than those in the secondary somatosensory area (S2). Moreover, this study demonstrated that S1 neuron responses to “in vitro” Po stimulation showed paired-pulse facilitation, small initial excitatory postsynaptic potentials (EPSPs), a graded activation profile, and a metabotropic receptor component, suggesting a modulatory role in information processing. In contrast, neurons recorded in S2 L4 showed paired-pulse depression, large initial EPSPs, an all-or-none activation profile, and no metabotropic receptor component, a profile typical of field-defining or “driver” inputs (Viaene et al. 2011a; reviewed by; Sherman and Guillery 2013).

To determine whether structural and/or functional differences exist between Po synapses in M1wk and S1BF, we first measured axonal length and varicosity distribution across cortical layers using stereological methods, and compared the size of Po synaptic boutons using both light and electron microscopies (EM). In addition, we examined the “in vivo” responses to Po electrical stimulation and pharmacologically investigated the glutamatergic receptor mechanisms involved. Our findings show that Po axon terminals are remarkably different both in structure and in functional impact in M1wk and S1BF.

## Methods

### Animals

Experiments were performed in adult (60–105 days old, 25–32 g in weight) male C57BL/6 mice. Five animals were used for axon tracing experiments (3 for light microscopy and 2 for EM) and 11 further animals for electrophysiological recordings. Animals were housed under standard colony conditions with food and water ad libitum under a 12-h light/dark cycle. All procedures involving live animals were conducted at the Autónoma de Madrid University under protocols approved by our University ethics committee and the competent Regional Government agency (PROEX175/16, PROEX189/16), in accordance with the European Community Council Directive 2010/63/UE. Efforts were made to minimize the number of animals required.

### Anesthetic procedures

All surgical procedures were conducted under isoflurane anesthesia (0.5–1% in oxygen) following induction with a combination of ketamine (0.075 mg/g body weight, i.p.) and xylazine (0.02 mg/g body weight, i.p.) for anatomical experiments or just a higher dose of isoflurane (3%) for electrophysiological recordings. During recordings, the appropriate level of anesthesia was monitored by the presence of delta frequency waves (1–4 Hz) of high amplitude (> 50 µV) in the field potentials, as well as by the absence of both spontaneous whisker movements and pinch withdrawal reflex. Buprenorphine hydrochloride (0.075 mg/kg body weight, s.c.) was administered for post-surgical analgesia in anatomical experiments. At the time of sacrifice, animals were overdosed with sodium pentobarbital (0.08 mg/g body weight, i.p.).

### BDA axonal tracing experiments

To anterogradely label the axons arising from populations of Po neurons, animals were positioned in a stereotaxic apparatus (David Kopf Instruments, Tujunga, CA, USA) and placed on a water-heated pad at 37 °C. The midline of the scalp was sectioned and retracted, and a small craniotomy was opened. Borosilicate glass micropipettes (1 mm outer diameter with internal glass filament; 10–20 µm of inner tip diameter; WPI, Sarasota, FL, USA) were loaded with a 3% solution of lysine-fixable biotinylated dextran amine (BDA) of 10,000 MW (Invitrogen, Carlsbad, CA, USA) in 0.01 M phosphate buffer (PB; pH 7.4) and stereotaxically positioned into Po following coordinates according to the Franklin and Paxinos (2008) mouse atlas (AP: − 1.8 mm, LM: 1.2 mm; DV: − 3 mm). A positive current of 2 µA (7 s on/off cycles) was applied for 10 min using a Midgard Current Source (Stoelting Co., Wood Dale, IL, USA). The micropipette was then left in place for additional 10 min before removal and wound closure. After awakening, animals were allowed to survive for 7 days after BDA injections and were then sacrificed as described below.

### “In vivo” electric thalamic stimulation and cortical recording

The animal was placed in a rodent stereotaxic frame as described above. After the scalp incision, lidocaine (1%) was applied locally. Three small craniotomies were made. The first over the M1wk which was identified based on the literature as the region where minimal intensity stimulation produces selective whisker movements in mice (Li and Waters 1991; Brecht et al. 2004; Hooks et al. 2011; Tennant et al. 2011). This first craniotomy was thus made along the border between area AgM/M2 and area AgL/M1 of the frontal cortex (AP: 0.5–1.5 mm, ML: 1–1.5 mm). The second craniotomy was made over S1BF (AP: from − 0.4 to − 1.8 mm, ML: 2.5–3.5 mm).

The third craniotomy was vertically located over Po (AP: − 1.8 mm, LM: 1.2 mm) according to the Franklin and Paxinos (2008) mouse atlas. A stainless steel bipolar electrode (120 µm diameter, WPI) was then vertically lowered into Po aiming at DV − 3.0 mm. Single square pulses were then delivered though the electrode (0.3-ms duration; Cibertec Stimulator, Madrid, Spain) to produce responses in S1BF and M1wk. Current intensity (20–80 µA) was adjusted in each experimental case to the double of the minimum required to elicit responses. When delivered through metal electrodes in a gray matter region of a mammalian brain, currents in the above range have been estimated to activate neuronal bodies within a maximal radius of less than 100–400 µm (Ranck 1975), respectively. Given the small dimensions of mouse Po, selective stimulation was performed by aiming the electrode to a central zone of the nucleus, and histologically verifying the electrode tip position after the experiments (Fig. 2b).

We applied a protocol of 50 single pulses at different frequencies (1, 5 and 10 Hz) separated by 20-s intervals without stimulation. Also, three paired-pulse stimulus paradigms were used in which four stimuli with the same current amplitude were delivered into Po with an inter-stimulus interval (ISI) of 50, 100 or 300 ms.

Unit recordings were performed through tungsten microelectrodes (2–4 MΩ; WPI) lowered stereotaxically into M1wk or S1BF. Units were recorded throughout the entire cortical depth. For analysis, data were pooled and compared between superficial (DV: 100–400 µm) or deep (DV: 500–1100 µm) cortical layers in each area. An unanalyzed gap of 100 µm was thus left between these compartments as a safeguard against potential errors in laminar localization due to small misalignments or drift of the electrode. In each experiment, we obtained two or three superficial layer recordings and two or three further recordings in deep cortical layers. Recordings were filtered at 0.3–3 kHz and amplified using a DAM80 preamplifier (WPI). Data were recorded continuously, sampled at 10 kHz, via an analog-to-digital converter built in to the Power 1401 data acquisition unit (Cambridge Electronic Design, Cambridge, UK) and digitally stored in a computer until further use. Mice were sacrificed with an overdose of anesthetics (see above) immediately after recordings were completed.

### Pharmacological blockade of glutamatergic receptors in the cortex

To examine the various glutamate receptor components involved in cortical responses to Po thalamocortical activation, the NMDA receptor antagonist D-2-amino-5-phosphonopentanoate (D-AP5; 50 µM; 0.1 µl; Biogen Científica, Madrid, Spain), the non-selective group I/group II metabotropic glutamate receptor antagonist (+)-alpha-methyl-4-carboxyphenylglycine (MCPG; 10 µM; 0.1 µl; Tocris Bioscience, Bristol, UK), and saline (control) were injected into M1wk and S1BF cortices in different animals. These were injected near the location of the tungsten microelectrode in each area (within a 500 µm radius to allow diffusion in the recording site). Drugs were slowly delivered through a cannula connected to a Hamilton syringe (1 µl; Bonaduz, Switzerland) over a 1-min period and recordings were performed 10 min after drug application.

### Perfusion and tissue processing for optical microscopy

Animals were first perfused transcardially with 6 ml of saline for 1 min, followed by 50 ml of 4% paraformaldehyde (PFA; diluted in 0.1 M PB, pH 7.4) for 8 min. Brains were then removed from the skull and postfixed overnight at 4 °C in the same fixative. Subsequently, brains were cryoprotected by soaking in 30% sucrose (0.1 M PB, 4 °C, 48 h). Two parallel series of coronal sections (60 µm thick) were obtained on a freezing microtome (SM 2400; Leica, Germany).

In the axonal labeling experiments, sections were processed using avidin–biotin–peroxidase (1:100; ABC, Vectastain Elite, Vector Laboratories, Burlingame, CA, USA) and diaminobenzidine–glucose oxidase, with nickel enhancement (Shu et al. 1988). Serial sections were alternatively counterstained with thionin or cytochrome oxidase histochemistry (CyO; Wong-Riley et al. 1979) to allow a precise cytoarchitectonic localization of the labeling. In the recording experiments, sections were stained only with CyO. Sections were finally mounted onto gelatin-coated glass slides, air dried, dehydrated through an ascending series of ethanol, defatted in xylene for 60 min and coverslipped with DePex (Serva, Heidelberg, Germany).

### Perfusion and tissue processing for EM

Seven days after the BDA injection, animals were perfused first with saline, and then with 4% PFA + 0.1% glutaraldehyde in 0.1 M PB for 30 min. Brains were then blocked and immersed for 1 h in 4% PFA in PB at 4 °C. Serial 50-µm-thick coronal vibratome sections (Leica VT 1200S) were collected in PB as two parallel series, and subsequently immersed in sucrose (30% in 0.1 M PB) overnight.

Both series of sections underwent three consecutive rapid freezing–thawing cycles in liquid nitrogen to increase membrane permeability, and were then processed, in parallel, to develop BDA as described above, with the exception that in the second series, the oxygen–peroxide blocking step and the nickel sulfate enhancement were omitted. The first series was used to localize labeled Po axon arborizations and BDA deposits. The parallel sections from the second series containing the region with the heaviest cortical labeling underwent further processing for EM. Free-floating sections were first incubated for 45 min in 1% osmium tetroxide in 0.1 M PB, washed in PB, rinsed in 50% ethanol, incubated for 40 min in 1% uranyl acetate diluted in 70% ethanol in the dark, and finally dehydrated in an ascending ethanol series to absolute ethanol. The dehydrated sections were transferred to acetonitrile, and then immersed in epoxy resin (Durcupan™; Electron Microscopy Science, Hartfield, PA, USA) overnight. Finally, sections were flat embedded in Durcupan™ and polymerized at 60 °C for 48 h. Under a dissection stereomicroscope, samples containing the appropriate layers in M1wk or S1BF cortices were cut out, and then glued onto pre-polymerized resin blocks.

Embedded tissue blocks were cut on an ultramicrotome (Leica Ultracut UCT) into ultrathin sections (~ 60 nm thickness) and collected on pioloform-coated slot copper grids. Thereafter, they were counterstained with lead citrate (3 min; Reynolds 1963).

### Stereological axon measurements

Stereological analyses were carried out with a BX61 light microscope (Olympus, Japan) equipped with a MT12 microcator (0.5-µm resolution; Heidenhain, Traunreut, Germany), a X–Y–Z high-precision motorized microscope stage (ProScan II; Prior Scientific, Cambridge, UK), and an Olympus DP-71 digital camera connected to a computer running the NewCAST stereology software package (Visiopharm, Hørsholm, Denmark).

In samples from three different Po axon-labeling experiments, absolute axonal length in individual cortical layers of S1BF and M1wk was estimated using the isotropic virtual plane method following a fractionator sampling scheme (Drøjdahl et al. 2010). For that purpose, the contours of the layers within the cortical column where the axonal arborizations were present were drawn for each cortical region with the 4x objective in the 60-µm-thick sections immunostained for BDA; then, these areas were sampled by the equidistant and parallel isotropic—randomly oriented—virtual planes which contain three-dimensional ‘sampling boxes’ inside. The intersections between the planes and the BDA-labeled fibers were counted. We used a distance between virtual planes of 20 µm, a sampling box volume of 47,020 µm^3^ and a step length area of between 10,000 and 22,500 µm^2^ (depending on the axonal density previously observed in each cortical layer). We considered and corrected the inhomogeneous shrinkage in the *z*-axis calculating the number-weighted mean section thickness, as previously described (Bermejo et al. 2003).

In addition, the absolute number of axonal varicosities in each layer was determined with the optical fractionator method (West and Gundersen 1990). Using the 100× oil-immersion objective in the same contours drawn for axonal estimations, swellings larger than twice the thickness of surrounding axon domains were identified as varicosities. Axon enlargements at branching points were not considered. The individual volume of the optical disectors used to sample the regions of interest (cortical layers) was 4704 µm^3^ and the step length area for each layer was the same as for axonal length estimations.

The distribution of axonal length and varicosities per layer was then calculated. The precision of the stereological estimations was calculated by obtaining the coefficient of error due to the sampling method (Gundersen et al. 1999). We aimed a minimum counting of 100 axonal intersections or varicosities per cortical layer to obtain a coefficient of error below 0.1. In only a few cases, the coefficient of error was higher than 0.1 because the areas were too small or axonal density too low to allow counting enough intersections or varicosities.

In addition, to facilitate comparison with the visual appearance in S1BF of Po arborizations in the radial dimension of the cortex, we calculated both “length density” (axonal length per volume) and “density of varicosities” (number of varicosities per volume) in each of the three cases included in the study. For this purpose, we estimated the volume of each layer across the cortical sector containing the entire Po-labeled axon population. This volume was estimated using the Cavalieri principle (Gundersen and Jensen 1987); a point grid with a known area per point was used and the number of points reaching each cortical layer in every section sampled were computed. The volume estimation was then obtained multiplying the number of points, the area per point and the separation between sections. Densities were obtained dividing the previously calculated absolute axonal length or number of varicosities by the volume in each layer.

### Light microscopy and EM bouton size analyses

Under bright-field optics in Nikon Eclipse 600 microscope (Nikon, Tokyo, Japan) the Po axonal arborizations appeared as sharply labeled filaments with frequent varicose swellings. To estimate and compare the size of varicosities (putative synaptic boutons), we measured the maximal projection area from live images using a Nikon DMX1200 camera fitted to the microscope and the NIS-Elements imaging software tools (v3.2; Nikon). To this end, the major perimeter of each varicosity was focused at 1000× and delineated over the computer screen using the polyline and polygon software tools. For each cortical area and for each animal, 500 randomly selected varicosities were measured. Three animals were analyzed (3000 varicosities in total). We did not include varicosities with cross-sectional areas (maximal projection) near or below the microscope resolution limit (< 0.2 µm^2^).

The size of Po axonal boutons was estimated and compared also from the area of sectioned profiles on EM micrographs. At the EM level, BDA-labeled Po boutons were easily identifiable by the electron-dense opaque DAB reaction product. For measuring the bouton size, only sections in which a synapse was visible were considered. Sections were examined with a Zeiss Libra 120 transmission electron microscope (Carl Zeiss, Oberkochen, Germany) equipped with a bottom-mounted Proscan 2K digital camera (Fa. Tröndle, Moorenweiss, Germany). Bouton profiles were analyzed on digital images obtained using the Multi-Image Acquisition software (SIS; Olympus, Münster, Germany) at a final magnification of 8000×. For each cortical region, the surface areas of 50 boutons were measured with OpenCAR software as described in detail in Rodriguez-Moreno et al. (2018).

### Electrophysiological data analysis

Single-unit activity was extracted with the aid of Spike2 software (Cambridge Electronic Design) for spike waveform identification and analysis. In some cases, antidromic activity was present, but these records were excluded from the analysis. The sorted spikes were analyzed and quantified using peri-stimulus time histograms (PSTH; bin width 1 ms), where time 0 ms corresponded to the onset for the first stimulus in the stimulation train. The PSTH was calculated from 50 successive Po electrical stimuli. Response magnitude was defined as the total number of spikes per stimulus occurring between stimulus onset and 50 ms afterwards. To analyze Po-evoked responses during the application of stimulation trains, we calculated the response magnitude variation as the percentage change of the spikes measured in the second (P2), the third (P3) and the fourth (P4) pulses with respect to the first pulse (P1; normalized as 100%).

Paired-pulse ratios (PPR) were defined as the cortical unit response to P2, P3 or P4 in any given train divided by the response to P1. Thus, a PPR of 1 reflects no change between pulses; PPR < 1 indicates depression of the response, and PPR > 1 indicates facilitation.

Initial (onset) and final (offset) latencies and response duration were measured in the PSTH. Initial latency was considered the time elapsed between stimulus onset and when the number of spikes doubled those in the spontaneous activity period. Final latency was considered the time when the number of spikes decayed to the same level as during the spontaneous activity period.

### Statistical analysis

Statistical analysis was computed using GraphPad Prism 5 software (San Diego, CA, USA) or SPSS Statistics software (v. 23; IBM, Armonk, NY, USA). The threshold level of significance was set at *p* < 0.05, indicating this as (*), *p* < 0.01 as (**) and *p* < 0.001 as (***).

For the stereological dataset, comparison was performed by one-way ANOVA and either Bonferroni or Games–Howell post hoc pair-wise comparison methods, depending on the homogeneity of variances. Varicosity size distribution and medians were compared between pairs of areas/layers with a two-sample Kolmogorov–Smirnov (K–S) test and the Mann–Whitney *U* test (M–WUT).

For the electrophysiological dataset, first we applied the Shapiro–Wilk normality test. Then, we used the paired Student’s two-tailed *t*-test or M-WUT, depending whether the data were normally distributed or not. For multiple comparisons, we used a two-way ANOVA, plus Bonferroni’s multiple comparison tests as a post hoc comparison method.

## Results

### The laminar distribution of Po varicosities in S1BF and M1wk is markedly different

**Fig. 1:**
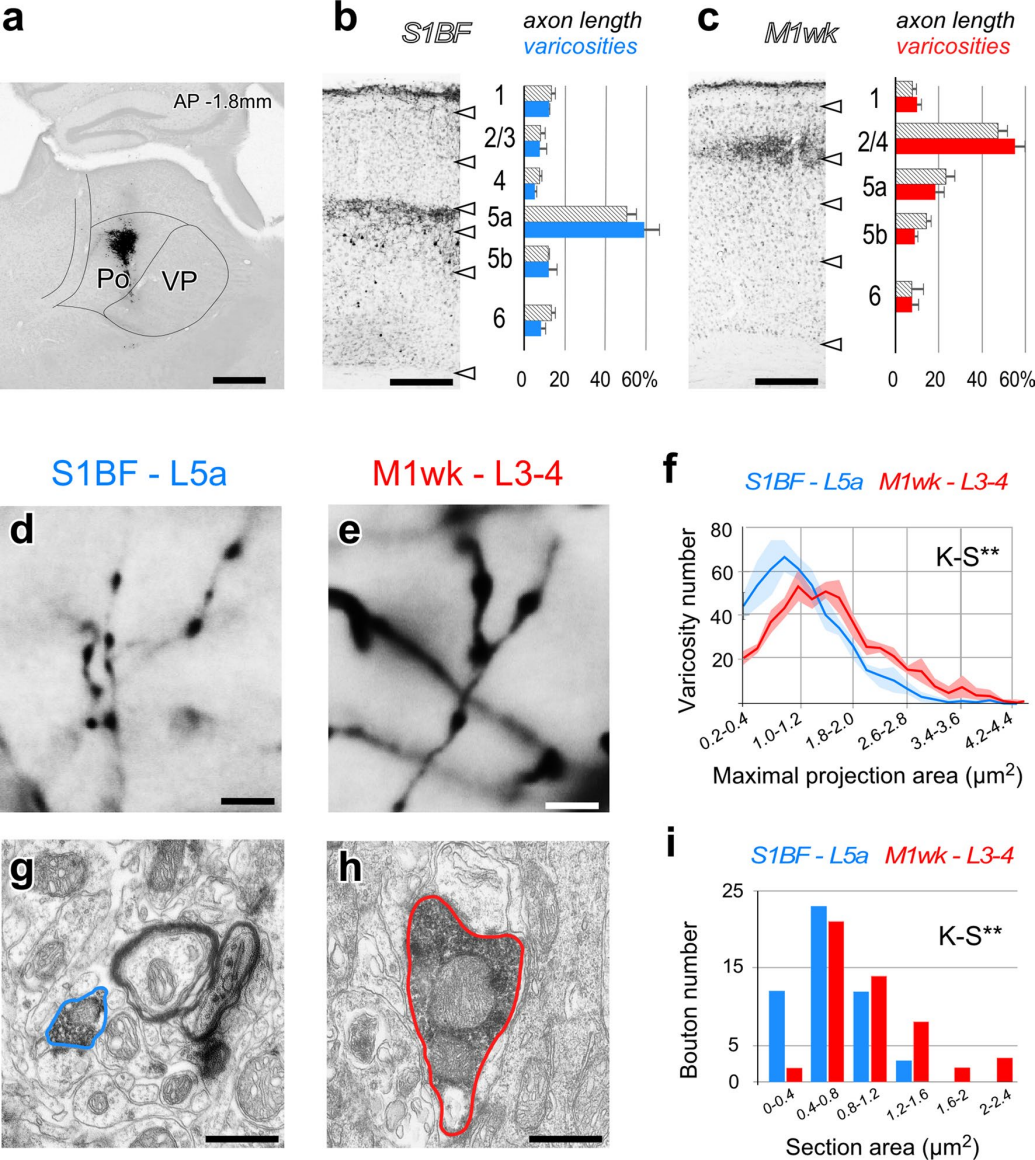
Quantitative analysis of axonal length, varicosity number and bouton size of Po axons in different layers of areas S1BF and M1wk. **a** Lightly thionin-counterstained coronal thalamus section showing the center of a representative iontophoretic BDA deposit in Po. The boundaries between Po and ventral posterior complex (VP) are indicated by contour lines. **b, c** Stereological estimates of axonal length (hatched bars) and number of varicosities (color bars) per individual cortical layer, as percentage of the total. For reference, a photomicrograph of the axonal BDA labeling in a representative coronal column sample from each area is provided (lightly thionin counterstain). High-magnification light-microscope images of BDA-labeled varicose axonal branches in L5a of the S1BF (“**d**”) and L3-4 of M1wk (“**e**”). Note the differences in the size of varicosities between the two areas. **f** Distribution of axon varicosity intervals (measured as maximal projection area) in M1wk L3–4 (blue line) or S1BF L5a (red line). SEM is represented as paler shadings of the same colors. Low-power EM images of BDA-labeled Po boutons in S1BF L5a (“**g**”) and M1wk L3–4 (“**h**”). Labeled bouton profiles are outlined in the same colors as above. **i** Range of Po bouton cross-sectional area measured on EM sections. Scale bars **a** 500 µm; **b, c** 250 µm; **d, e** 5 µm; **g, h** 1 µm

To examine possible structural differences between Po axonal terminals in S1BF and M1wk, we selectively labeled Po populations of axons by means of iontophoretic BDA injections in the thalamus. Three experiments in which a tracer deposit was confined to Po were analyzed (Fig. 1a). The glucose oxidase BDA–nickel labeling protocol produced a sharp, continuous, Golgi-like axonal labeling. In all injections, axonal arborizations with varicose swellings were clearly identifiable at the light-microscope level (Fig. 1a–f).

In M1wk and S1BF, a marked layer-specific distribution of axonal arborizations was observed (Fig. 1b, c). In general, axonal varicosities at thalamocortical axons, most of which represent synaptic sites (White et al. 2004; Rodriguez-Moreno et al. 2018), were obscured by the mess of labeled axon filaments. Thus, we separately quantified axonal lengths and varicosity numbers with stereological methods in all cortical layers, and compared them between the two areas (Fig. 1b, c; Supplementary materials Table SM1). The axonal length analysis confirmed that while the mean length was virtually identical in layers 1, 5b and 6, it was significantly higher by nearly twofold in L5a of S1BF (48% ± 7.7) when compared with M1wk (23.3% ± 7.2; *p* = 0.006). Conversely, axonal length was lower by threefold in L2–4 of S1BF (14.9% ± 5.7) than in the equivalent layers of M1wk (47.6% ± 7.7; *p* = 0.004). Differences in the layer-specific distribution of axonal varicosities were even more striking: 56.2% ± 11.2 were located in L5a of S1BF but only 18.4% ± 7.1 were observed in the same layer of M1wk (*p* < 0.001). Conversely, while just 12.5% ± 6.4 Po varicosities were located in L2–4 of S1BF, the equivalent layers of M1wk contained 55.5% ± 8.1 (*p* < 0.001). The sharp inversion observed in the distribution of Po axonal varicosities in L2–4 and L5a of the two areas seems all the more significant in view that distributions in the remaining cortical layers were similar.

### Po axonal boutons in M1wk are significantly larger than those in S1BF

Using high-power light microscopy, Po axonal varicosities in M1wk looked substantially larger than those in S1BF (Fig. 1d, e). To quantitatively assess these differences, we first compared the size of axonal varicosities (maximal projection areas measured at 1000×; 500 varicosities per experiment, in three experiments; see Methods). We demonstrate that, on average, Po varicosities in M1wk L3–4 were significantly larger (1.6 ± 0.8 µm^2^) than in those in S1BF L5a (1.01 ± 0.5 µm^2^; M–WUT, *p* < 0.001), and had a significantly different frequency in size distribution (K–S, *p* < 0.001; Fig. 1f) with a substantial tail of larger boutons.

To further quantify and distinguish between these two populations, we also measured bouton size on a sample of EM single images of BDA-labeled Po synaptic boutons. Surface area measurements confirmed that M1wk L3–4 boutons were significantly larger than those in S1BF L5a (0.99 ± 0.5 µm^2^ vs. 0.67 ± 0.3 µm^2^; M–WUT, *p* < 0.001). Moreover, Po synaptic boutons had a different frequency of size distribution in the above two areas (K–S, *p* = 0.009; Fig. 1i). In addition, this analysis showed that, in general, axonal varicosities correspond to asymmetric synaptic sites (Fig. 1g, h) indicative of their glutamatergic nature.

### Po synapses in S1BF and M1wk show different dynamic profiles “in vivo”

**Fig. 2:**
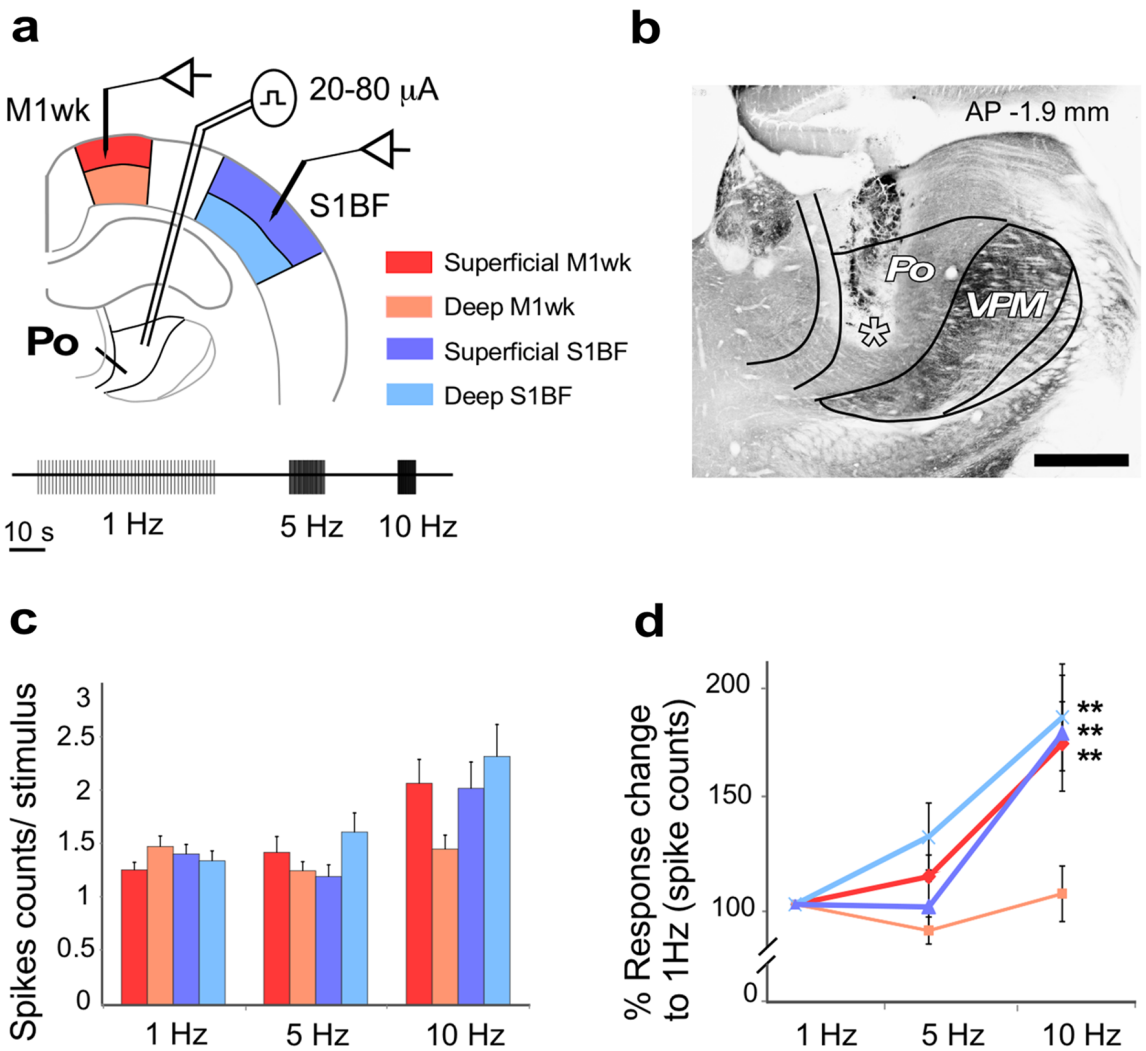
M1wk and S1BF unit responses to Po “in vivo” stimulation at different frequencies. **a** Schematic drawing of the experimental design. Electrical pulses (0.3 ms, 20–80 µA) were applied to Po and unit responses were recorded in S1BF or M1wk cortices. Units recorded in superficial or deep cortical layers were indicated by different colors. A protocol of 50 single pulses delivered at three different frequencies (1, 5 and 10 Hz) separated by 20-s pause intervals was applied. **b** Representative coronal section of the thalamus showing the trace of the bipolar electrode in Po. Tip position is indicated by an asterisk. Cytochrome oxidase counterstain. Bregma anteroposterior level (AP) is indicated. VPM: ventral posteromedial nucleus. Bar 500 µm. S1BF and M1wk unit responses to Po stimulation at different frequencies. In “**c**”, spike counts per stimulus are compared. In “**d**”, the percent of change at 5 and 10 Hz is compared to values recorded at 1 Hz

In several glutamatergic projection systems, including thalamocortical axons, differences in synaptic bouton size have been correlated with specific functional properties (reviewed in Sherman and Guillery 2013). These include amplitude changes to repetitive stimulation and the involvement of different receptor mechanisms. To investigate possible functional differences in M1wk and S1BF unit responses to repetitive Po activation, trains of electrical stimulation to Po were applied and recordings were obtained extracellularly in superficial and deep cortical layers of each area (Fig. 2a, b). Spike counts revealed a low spontaneous activity in both superficial and deep neuronal populations in M1wk (0.6 ± 0.1 spikes/s, *n* = 33; and 0.4 ± 0.1 spikes/s; *n* = 23, respectively) and in S1BF (0.5 ± 0.1 spikes/s, *n* = 32; and 0.6 ± 0.1 spikes/s; *n* = 21, respectively).

First, we examined M1wk and S1BF unit responses to electrical Po “in vivo” stimulation at increasing frequencies (1, 5 and 10 Hz; 20–80 µA; Fig. 2a). Single-pulse Po stimulation induced orthodromic spike responses in all layers of M1wk and S1BF (Fig. 2c). 1 Hz single-pulse Po stimulation elicited 1.3 ± 0.1 spikes/stimuli in superficial layers and 1.5 ± 0.1 in deep layers of M1wk and 1.4 ± 0.1 in both S1BF superficial and deep layers. However, no significant differences in spike probability were observed between M1wk and S1BF in either superficial (*p* = 0.193) or deep layers (*p* = 0.327). Units in M1wk superficial layers increased their firing at higher stimulation frequencies (*F* = 6.68, *p* = 0.002), while those in deep layers did not change (*F* = 1.26; *p* = 0.292; Fig. 2d). In S1BF, neurons recorded in both the superficial and the deep layers increased their firing at higher Po stimulation frequencies (*F* = 6.42, *p* = 0.002 and *F* = 5.40, *p* = 0.007, respectively; Fig. 2d).

**Fig. 3:**
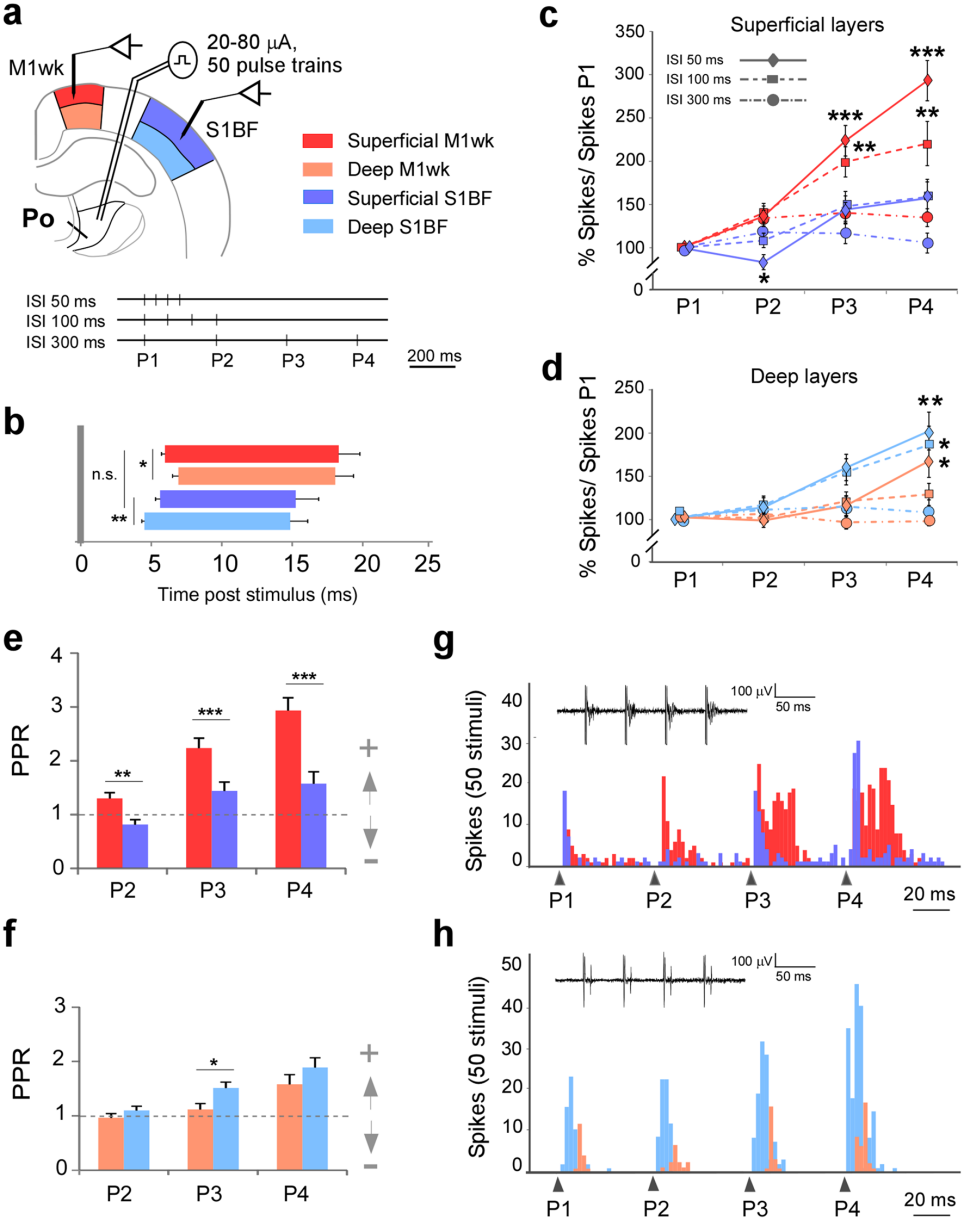
M1wk and S1BF unit responses display markedly different temporal profiles to repetitive Po stimulation. **a** Experimental design. Electrical pulses were applied to Po and unit responses were recorded in the cortex. Three different electrical stimulation protocols were applied, consisting each of 50 trains of current pulses delivered every 3 s. Four pulses (P1–P4) were delivered; for each protocol the pulses were spaced at progressively longer intervals (ISI; 50, 100 or 300 ms) between them. **b** Unit response latencies and durations to Po electrical stimulation (P1, 50 stimuli). Mean onset and offset latencies are represented as horizontal bars. The vertical gray bar indicates stimuli application. **c**, **d** Changes in spike counts are represented as the percentage of spikes counted after each pulse (P1–P4) divided by spikes after P1. Counts obtained in each of the above three protocols are represented by lines with different dashes. Counts in superficial (“**c**”) or deep (“**d**”) layers in both areas are shown. Color coding as in panel “**a**”. **e, f** Paired-pulse ratios (PPR) represented as spike counts after every pulse divided by spike counts in the first pulse (P1). **g**, **h** An example of M1wk and S1BF unit responses with the 50-ms inter-pulse protocol. Peri-stimulus time histogram (PSTH) represents spike counts in superficial (“**g**”) and deep (“**h**”) layers. Arrowheads indicate pulse application. Representative spike traces are shown inside PSTHs

In addition, we applied several protocols, each using a different frequency of repetitive Po stimulation to examine short-term changes in unit responses. Four pulses (P1–P4) spaced at different intervals (ISI) of 50, 100 or 300 ms were applied in each protocol (Fig. 3a).

Besides receiving inputs from Po, M1wk and S1BF are known to be heavily linked by cortico-cortical connections (Mao et al. 2011; Hooks et al. 2013). As a way to differentiate between the direct effects of Po inputs to both areas vs. the effect of connections between the areas, we examined the onset and offset latencies and the response duration to P1 in ISI 50-ms protocol (Fig. 3b). This analysis showed that the first activation (onset latency) in M1wk and S1BF was almost simultaneous (*p* = 0.631). First responses consistently appeared in S1BF deep layers 4.6 ± 0.2 ms after Po stimulation (*p* = 0.005), then 5.6 ± 0.3 ms in S1BF superficial layers and 5.8 ± 0.3 ms in M1wk superficial layers. Finally, responses occurred with a slightly longer latency in the deep layers of M1wk (6.9 ± 0.4 ms; *p* = 0.027). No differences were found in response durations between M1wk and S1BF (*p* = 0.089 in superficial layers, *p* = 0.329 in deep layers).

The above four-pulse Po stimulation trains elicited a marked facilitation of unit responses in the superficial layers of M1wk (Fig. 3c). In each train, spike count increases were evident already for the second pulse (P2), and became even more pronounced following the third (P3) and fourth (P4) pulses. At 50-ms inter-stimuli intervals (*F* = 15.17, *p* < 0.001) spikes at P4 increased by nearly threefold when compared with spikes counted after P1 (*p* < 0.001). A weaker facilitation effect was observed with 100-ms inter-stimuli intervals (*F* = 5.97; *p* = 0.010). No change was observed when pulses were separated by 300 ms (*F* = 1.54, *p* = 0.207). In contrast, using the same Po stimulation protocol (ISI 50 ms), unit responses were significantly decreased after P2 in S1BF superficial layers (a decrease from 1.3 ± 0.13 spikes/stimuli in P1 to 1.0 ± 0.12 in P2; *p* = 0.047), and no changes were observed after P3 or P4, nor during the 100 or 300 ms ISI protocols (Fig. 3c). Statistical comparison between response changes in superficial layers of M1wk and S1BF during the 50-ms and the 100-ms ISI protocols further indicated that, as a population, neurons in each area responded in significantly different ways (M-WUT, *p* < 0.001 and *p* = 0.049, respectively).

Although less striking, changes in spike counts during the four successive pulses of Po stimulation were also observed in the deep layers of both areas (Fig. 3d). In M1wk, only a small facilitation of the P4 response with the 50-ms ISI protocol was observed (*F* = 2.78, *p* = 0.046). In S1BF, response facilitation was observed with both 50- and 100-ms ISI protocols (*F* = 6.22, *p* = 0.001 and *F* = 3.82, *p* = 0.013, respectively). Statistical comparison between response changes in deep layers during the 100-ms ISI protocol demonstrated that neurons in each area, as a population, displayed significantly different temporal response profiles (*p* = 0.026).

Analysis of average paired-pulse ratios for the ISI 50-ms protocol confirmed the statistical significance of the above differences (Fig. 3e, f). Examples of representative M1wk and S1BF cell recordings are shown in Fig. 3g (superficial layers) and 3 h (deep layers). In superficial layers (Fig. 3e), this ratio was higher in M1wk than in S1BF in all single pulses (P2, *p* = 0.001; P3 and P4, *p* < 0.001). Remarkably, in S1BF superficial layers the ratio was 0.8 ± 0.09 in P2, indicative for depression of the response. In deep layers of both areas (Fig. 3f), this ratio is higher in S1BF (P3, *p* = 0.013) and all ratios indicated facilitation.

### Different glutamate receptor types mediate responses in Po synaptic boutons in S1BF and M1wk

**Fig. 4:**
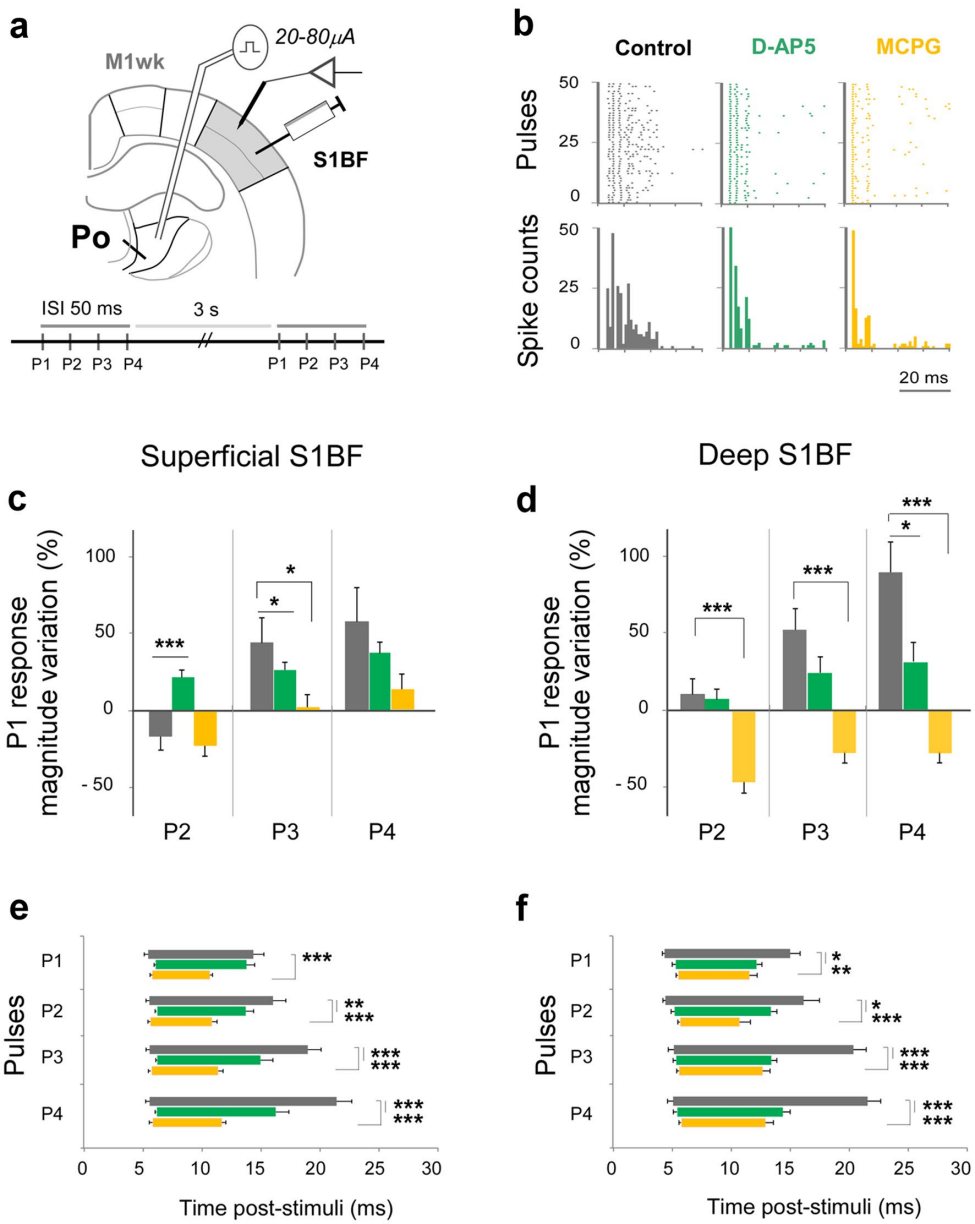
Pharmacological analysis of glutamatergic receptor systems involved in the “in vivo” S1BF responses to Po activation. **a** Experimental design. Ten minutes after an injection in S1BF of either the NMDA receptor antagonist D-AP5, the metabotropic receptor antagonist MCPG or a saline control, a four-pulse stimulation protocol (ISI 50 ms) was delivered to Po, and unit responses were recorded in the injected area. **b** Three representative single-unit response profiles (superficial layers of S1BF). Raster plots (top row) or PSTH (bottom) obtained under each drug condition. Gray: saline; green: D-AP5; yellow: MCPG; the same color coding is used in the panels below. Unit response variation in S1BF superficial (“**c**”) or deep (“**d**”) layers represented as the difference (%) between spikes after P2, P3 and P4 compared with those counted after P1. **e**, **f** Latency and duration of unit responses in superficial (“**e**”) and deep (“**f**”) S1BF layers. Post-stimulus intervals between mean onset and offset latencies are represented as horizontal bars

**Fig. 5:**
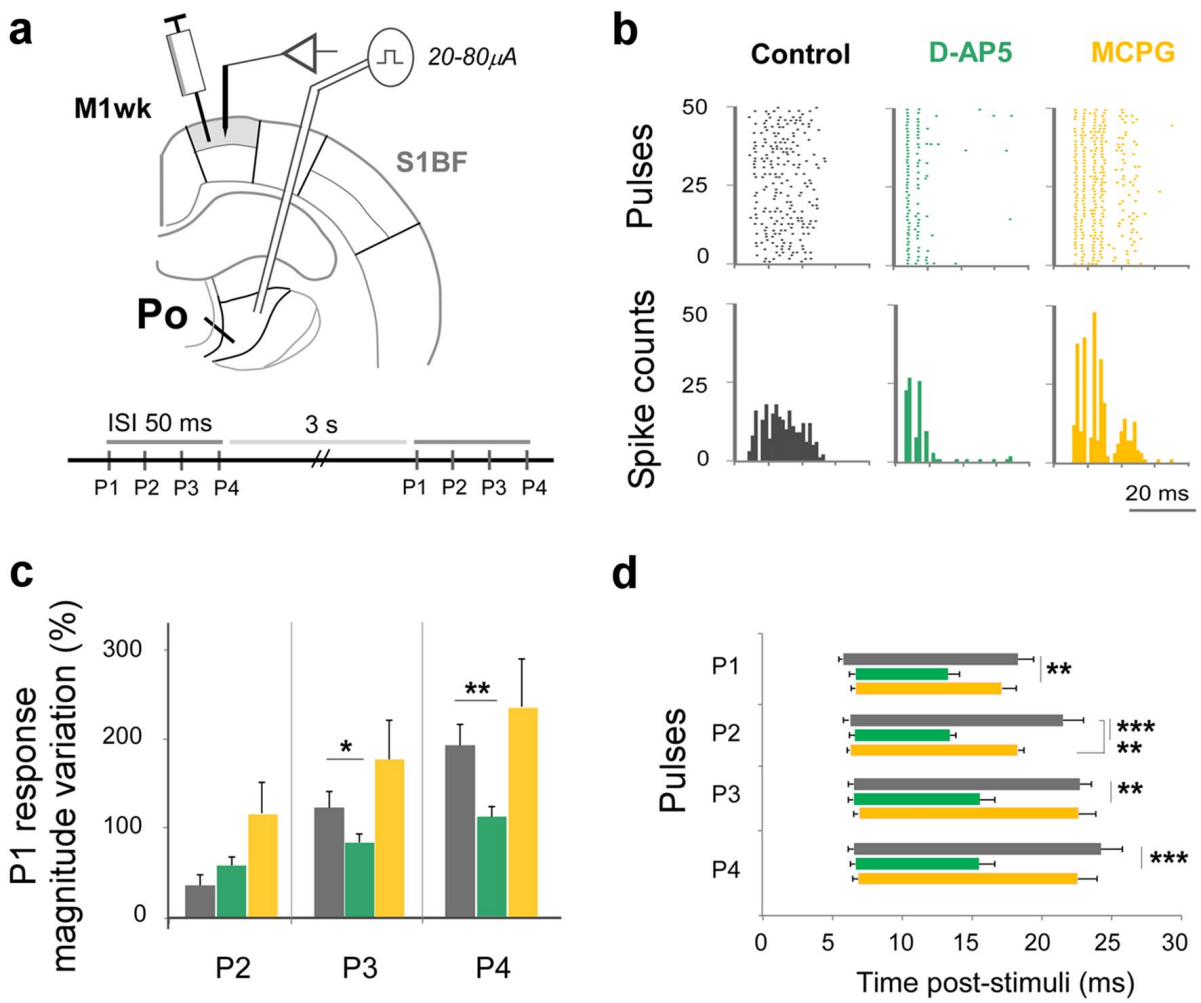
Pharmacological analysis of glutamatergic receptor systems involved in “in vivo” M1wk responses to Po activation. **a** Experimental design. Ten minutes after an injection in M1wk D-AP5, MCPG or saline (control), a four-pulse stimulation protocol (ISI 50 ms) was delivered to Po, and unit responses were recorded in the injected area. **b** Three representative single-unit response profiles in M1wk superficial layers. Raster plots (top row) or PSTH (bottom) obtained under each drug condition. Gray: saline; green: D-AP5; yellow: MCPG; the same color coding is used in the panels below. **c** Unit response variation in M1wk superficial layers. Variation is represented as the difference (%) between spikes after P2, P3 and P4 compared with those counted after P1. **d** Latency and duration of unit responses in superficial M1wk layers elicited by Po activation. Post-stimulus intervals between mean onset and offset latencies are represented as horizontal bars

To investigate whether the different response profiles of S1BF and M1wk cells reflect the involvement of specific glutamate receptor types at Po synaptic boutons in each area, the effect of different glutamate receptor antagonists on unit response profiles was performed. Using the ISI 50-ms train stimulation protocol, responses in each area after a local injection of saline (control), D-AP5 (a competitive NMDA antagonist) or MCPG (a non-selective group I/group II metabotropic glutamate receptor antagonist; Figs. 4a, 5a) were compared.

In S1BF (Fig. 4), the facilitatory effects to Po stimulation observed in controls were significantly reduced by D-AP5 in both superficial (P3, *p* = 0.013; *n* = 24; green columns in Fig. 4b, c) and deep (P4, *p* = 0.034; *n* = 14; Fig. 4d) cortical layers. Moreover, D-AP5 reversed the spike count decrease observed in P2 in S1BF superficial layers (*p* < 0.001; Fig. 4c). D-AP5 also reduced response duration in both superficial (P2, *p* = 0.007; P3 and P4, *p* < 0.001; Fig. 4e) and deep layers (P1, *p* = 0.011; P2, *p* = 0.034; P3–P4, *p* < 0.001; Fig. 4f). Likewise, responses in S1BF were strongly affected by MCPG. In superficial layers the facilitation observed in control condition was reduced (P3, *p* = 0.039; *n* = 28; yellow columns in Fig. 4b, c). In deep layers, MCPG completely reversed facilitation, producing a marked decrease of the responses after all pulses (P2–P4, *p* < 0.001; *n* = 15; Fig. 4d). Response duration was also shortened by MCPG (superficial layers P1–P4, *p* < 0.001; Fig. 4e; and deep layers P1, *p* = 0.005 and P2–P4, *p* < 0.001; Fig. 4f). Taken together, these observations strongly suggest that both ionotropic NMDA and metabotropic glutamate receptors are involved in response behaviors to Po inputs in S1BF.

In M1wk (Fig. 5), the robust facilitation of cell firing in superficial layers elicited by repetitive Po stimulation observed in controls was partially blocked by D-AP5 (P3, *p* = 0.037; P4, *p* = 0.004; *n* = 32; green columns in Fig. 5b, c). Responses after every pulse were shortened (P1, *p* = 0.006; P3, *p* = 0.001; P2 and P4, *p* < 0.001). However, no spike count changes were observed following the application of MCPG in this area (P2, *p* = 0.452; P3, *p* = 0.714; P4, *p* = 0.528; *n* = 28; yellow columns in Fig. 5b, c). Moreover, no consistent changes were observed in response duration (only a small decrease after P2, *p* = 0.005; Fig. 5d). Overall, these observations seem to indicate that ionotropic NMDA receptors mediate the responses to Po activation in M1wk and that metabotropic glutamate receptors play virtually no role in them.

## Discussion

Our results reveal striking differences between the vibrissa-related domains of the motor and primary somatosensory cortices regarding the layer distribution and size of thalamocortical Po synaptic boutons, the dynamic response profiles of cortical neurons to Po activation, and the glutamate receptor types that mediate these responses.

### Po synaptic inputs to the vibrissal motor or sensory cortices preferentially target different layers

The percent distribution of Po axonal length in S1BF we report here is congruent with our previous observations in a study that examined changes in response to infraorbital nerve lesions (Frangeul et al. 2014). It is also overall consistent with previous non-quantitative observations in rat using bulk (Herkenham 1986; Koralek et al. 1988; Chmielowska et al. 1989; Lu and Lin 1993; Wimmer et al. 2010) or single-axon anterograde labeling methods (Deschênes et al. 1998; Noseda et al. 2011; Ohno et al. 2012) which noted that Po axons in S1BF arborize preferentially in layers 5 and 1. Our data indicate that a large majority of Po arborizations in S1BF are located in L5a. The appearance of heavy axon labeling in L1 of S1BF (Fig. 1b, see also Meyer et al. 2010) thus seems to reflect, to a large extent, the tight packing of a relatively few axonal branches within the small volume of subpial layer 1. To confirm this interpretation, we calculated axonal length density (length/volume) in S1BF for each of the three BDA labeling experiments analyzed in this study. This revealed evident density peaks in both L5a and L1 in all cases (Supplementary Materials Fig. SM1). Besides, it is to be noted that our BDA deposits were limited to the rostral half of Po, because this is the region that preferentially innervates M1wk. The single-neuron axon arborization measurements in rats of Ohno et al. (2012) have shown that rostral Po neurons arborize mostly in L5a, while caudal Po/triangular nucleus neurons arborize preferentially in L1. Studies of Po axon distribution in rodent motor cortex (Herkenham 1986; Deschênes et al. 1998; Ohno et al. 2012; Yamawaki et al. 2014) are consistent with our finding that layers 4–3 are the main target of Po axons (Fig. 1; Supplementary Materials Table SM1).

The number and layer distribution of axonal varicosities in thalamocortical axons are of functional relevance, as varicosities have been shown to correlate closely with synaptic sites in these axons (White et al. 2004; Rodriguez-Moreno et al. 2018). Here, we demonstrate that more than half of Po synaptic boutons in S1BF are located in L5a, and less than 13% in L1. As with axon branch lengths, the analysis of the density of varicosities in S1BF revealed also a peak in L1, since the Po axonal arborizations in this layer are spatially concentrated in a narrow subpial band (Fig. SM1). These results are in agreement with a densitometric fluorescence estimation of Po synapse numbers across a S1BF column of GFP-tagged synaptophysin (Meyer et al. 2010), as well as with the finding that S1BF L5 neurons are much more powerfully excited by the selective optogenetic activation of Po axon terminals in L5a than by those in L1 (Petreanu et al. 2009). No study had previously examined Po axon varicosity distribution in M1wk.

Given the highly layer-specific arrangement of neurons and dendrites in the neocortex, the observation that Po axons exhibit marked differences in their layer distribution strongly suggests possible differences in the postsynaptic targets of these axons between S1BF and M1wk. It must be noted that the cortico-cortical “feed-forward”/“bottom-up” S1BF axons innervating M1wk arborize in roughly the same layers than Po synaptic boutons do (Mao et al. 2011; Petrof et al. 2015; Hooks et al. 2015). In turn, the M1wk to S1BF cortico-cortical axons, which are thought to provide “feedback”/“top-down” information, arborize in largely the same layers than Po synaptic boutons in S1BF (Johnson and Burkhalter 1996; Crick and Koch 1998; Mao et al. 2011). The contrasting laminar distribution of Po axons in M1wk and S1BF might, therefore, reflect a more general wiring pattern of cortical circuits, and be related to the different hierarchical levels of each area within the cortico-thalamo-cortical information-processing loops that control whisking (Ahissar and Oram 2015).

### Po synaptic boutons are larger in the vibrissal motor cortex than in the somatosensory barrel cortex

Using both light and electron microscopies, we demonstrate that Po synaptic boutons in S1BF L5a are, on average, significantly smaller than those in M1wk L3–4. Previous light microscopic estimations of Po varicosity sizes either in S1BF (labeled using BDA-ABC after a BDA injection in Po; Viaene et al. 2011a) or in M1 (labeled using GFP florescence after a viral vector injection in Po; Mo and Sherman 2019) are consistent with our observations. It is also interesting to note that present light microscopic measurements of maximal projection area of Po varicosities in L3–4 M1wk (1.6 ± 0.8 µm^2^) are close to that of our VPM thalamocortical varicosities in the L4 S1BF barrels (1.8 ± 0.8 µm^2^; Rodriguez-Moreno et al. 2018). Moreover, the finding that M1wk L4 Po boutons are comparatively large is unexpected, because motor cortex L4 thalamocortical boutons (which arise from both Po and from the ventrolateral nucleus) reportedly are, as a whole, smaller than L4 of S1BF VPM boutons (Bopp et al. 2017).

Studies in different glutamatergic forebrain projection systems, including thalamocortical axons, have shown that bouton size correlates with specific structural and functional synaptic properties. Larger synaptic boutons contain more mitochondria contributing to a larger mitochondrial volume, larger presynaptic vesicle pools, and more extensive and complex active zones (Rollenhagen et al. 2015; Bopp et al. 2017, Hsu et al. 2017; Rodriguez-Moreno et al. 2018; Yakoubi et al. 2018). These features all contribute to increase synaptic neurotransmitter release probability and strength (Matz et al. 2010; Holderith et al. 2012; Bourne et al. 2013; Rollenhagen et al. 2018). As a result, signal transduction in larger synaptic boutons may be both more effective and temporally more precise than that in smaller boutons (Sherman and Guillery 2002, 2013).

### Po synapses in S1BF and M1wk show marked differences in functional dynamics

Low-intensity electrical stimulation of the rodent thalamus as used here has been shown to activate cortical neurons through thalamocortical synapses (Rose and Metherate 2001; Castejon et al. 2016). Po nucleus stimulation elicited similar orthodromic responses in superficial and deep layers of both M1wk and S1BF. These findings are consistent with “in vitro” (Bureau et al. 2006; Petreanu et al. 2009; Viaene et al. 2011a) and “in vivo” (Gambino et al. 2014; Jouhanneau et al. 2014; Castejon et al. 2016; Mease et al. 2016) observations showing that rodent Po synapses elicit EPSPs in and/or spikes in S1BF pyramidal cells. Moreover, we show that mouse Po neurons are also able to drive cells in superficial and deep M1wk layers, registered with recent rat “in vivo” and mouse “in vitro” studies (Casas-Torremocha et al. 2017; Hooks et al. 2013; Mo and Sherman 2019).

Remarkably, we found that first-unit responses elicited by Po stimulation in S1BF and M1wk are virtually simultaneous. Unit activity in S1BF appears only 1.3 ms earlier than in M1wk. This minimal delay is compatible with a direct monosynaptic effect in both areas, considering that thalamocortical axons are fast conducting (~ 1 m/s) and that the two areas are at least 3 mm apart (Franklin and Paxinos 2008). Some previous studies in rat using sensory stimuli suited to drive Po cells (multi-whisker mechanical or whisker pad electrical stimulation; Farkas et al. 1999; Chakrabarti et al. 2008; Casas-Torremocha et al. 2017) noted that responses in M1wk and S1BF showed similar time courses. In contrast, the controlled mechanical stimulation of a single-whisker (which selectively activates VPM axons, but not Po axons) produces first a wave of activity in S1BF and only 8 ms later a wave of activation in M1wk, which is mediated by cortico-cortical connections (Ferezou et al. 2007).

The near-simultaneity of the first responses in M1wk and S1BF is entirely consistent with single-cell anatomical observations showing that Po signals may reach different cortical areas thought branched axons (Deschênes et al. 1998; Ohno et al. 2012) although it is not yet clear to which extent structurally different Po synaptic boutons arise from specific subpopulations of Po cells or from branched axons of the same neurons targeting both areas (Clasca et al. 2016).

In addition, we show that the neuron response profiles are markedly different in superficial vs. deep layers of both areas. Superficial neurons of M1wk and S1BF, as well as deep S1BF cells increase their firing rate to current pulse trains delivered at progressively higher frequencies, while M1wk deep neurons do not change their spiking behavior (Fig. 2). Likewise, repetitive single-pulse stimulation reveals divergent dynamic response profiles (Fig. 3) resulting in a strong facilitation in M1wk superficial layers but not in S1BF. Firing rates in superficial S1BF neurons even show a significant decrease after the second pulse. Such area- and layer-specific shifts in the global excitation/inhibition balance may reflect several factors. The larger glutamatergic boutons in M1wk are likely to have higher release probability (Viaene et al. 2011a; Ermolyuk et al. 2012; Bourne et al. 2013; Bickford 2016; Rodriguez-Moreno et al. 2018). Moreover, there is recent electrophysiological evidence that Po boutons target specific types of GABAergic interneurons in different layers in S1BF (Castejon et al. 2016; Audette et al. 2018; Williams and Holtmaat 2018). Thus, differences in synaptic bouton distribution across cortical layers may imply different interactions in each area. For example, recent EM data suggest that thalamocortical synapses onto GABAergic interneurons in L4 of the motor cortex may be rare or absent (Bopp et al. 2017).

On the other hand, adaptation is an ubiquitous property of forebrain sensory-motor feedback loops (Ahissar et al. 2000; Maravall et al. 2007). Response depression to repetitive stimulation of sensory organs is the most common form of adaptation observed in these loops. In the somatosensory system, most studies observed robust depression in different layers of S1BF to passive single and multi-whisker stimulations (Ahissar et al. 2000, 2001; Chung et al. 2002; Martin-Cortecero and Nuñez 2014); however, response facilitation has been reported in some studies (Brecht and Sakmann 2002; Garabedian et al. 2003). By comparing S1BF unit responses to electrical activation of the facial nerve that moves the whiskers (“active whisking”) vs. the same paradigm of “active whisking” and an additional contact of the vibrissa with an object (“touch”), Derdikman et al. (2006) concluded that adaptation in S1BF is both layer and stimulus specific. For example, responses in L4-barrels were facilitated by “touch”, while those in L5a were facilitated in both “touch” and “whisking” conditions and in L2–3 were depressed in both conditions. This is interesting in view of the anatomical and physiological evidence that the various types of whisker-related signals reach the cortex though largely parallel transthalamic pathways: the whisking signals via Po (“paralemniscal”), contact signals via ventrolateral portion of VPM (“extralemniscal”) and complex whisking-touch signals via dorsomedial portion of VPM (“lemniscal”; Yu et al. 2006).

Despite the fact that direct electrical Po stimulation and peripheral sensory receptor stimulation are not directly comparable, we observed different forms of adaptation between different S1BF layers. This pattern is reminiscent of that produced by “whisking” in this area (Derdikman et al. 2006). Adaptation in M1wk was also different from that observed in S1BF. Overall, these differences suggest that the effects of Po synapses on processes such as gain control or optimization of information transmission may be area-specific (Fairhall et al. 2001; Kohn and Whitsel 2002).

In addition, the observation that the effects of Po activation are more pronounced in M1wk neurons is consistent with our previous findings that silencing Po with muscimol drastically reduce evoked potential responses to whisker sensory stimulation in M1wk, while these responses in S1BF remain nearly unchanged (Casas-Torremocha et al. 2017) and unit firing rate increases (Castejon et al. 2016).

### Po synapses in S1BF and M1wk are mediated by different glutamate receptor types

We found that the facilitation elicited by Po stimulation in superficial M1wk layers is partially blocked by the NMDA receptor antagonist D-AP5, but not by the non-selective metabotropic receptor antagonist MCPG. The late component was the most affected, shortening total response duration (Fig. 5c), a finding consistent with NMDA receptors mediating the long-lasting response enhancement to repetitive stimulation (Daw et al. 2006; Remy and Spruston 2007). By contrast, Po-evoked facilitation in S1BF is shortened and partially blocked by both D-AP5 and MCPG (Fig. 4). This supports “in vitro” observations demonstrating the involvement of both receptors in S1BF responses to Po activation (Viaene et al. 2011a).

NMDA receptors participate in the late-lasting component of the S1BF responses to whisker sensory stimulation (Armstrong-James et al. 1993; Banerjee et al. 2009; Barros-Zulaica et al. 2014). Moreover, they have been recently shown to mediate Po-dependent long-term potentiation of S1BF neurons following repetitive whisker stimulation (Gambino et al. 2014; Williams and Holtmaat 2018). In addition, recent studies have begun exploring the role of NMDA receptors in motor cortex (Hasan et al. 2013; Kida and Mitsushima 2018). Our “in vivo” experimental protocol using wild-type animals is not adequate to block the pre- or postsynaptic effect to NMDA on responses to Po stimulation. Thus, we cannot resolve between pre- and postsynaptic NMDA effects. It has to be noted that NMDA presynaptic receptors are actually present in layer L4–L2/3 mouse somatosensory cortex synapses (Bender et al. 2006; Brasier and Feldman 2008; Banerjee et al. 2016).

The observed shortening of S1BF responses produced by MCPG is consistent with an involvement of metabotropic receptors in long-lasting response modulation (reviewed in Sherman 2004; Chung and Kim 2017).

Glutamatergic axon terminals have been classified into two broad functional categories according to specific combinations of structural and functional traits (for review see Sherman and Guillery 2013). The first are large terminals with extensive active zones and vesicle pools, in which ionotropic receptor-mediated transmission produces large initial EPSPs, paired-pulse depression and an all-or-none activation profile (“class 1” or “driver” synapses). Terminals of the second category (“class 2” or “modulatory”) are smaller and/or have comparably smaller active zones and vesicle pools. They involve both ionotropic and metabotropic receptors, and elicit small EPSPs that show paired-pulse facilitation and graded activation. Distance to the cell soma and/or spatiotemporal convergence may further modify the functional impact of such synapse types (Bruno and Sakmann 2006; Viaene et al. 2011b). Overall, class 1 terminals can “drive” postsynaptic neurons and strongly affect their output, while class 2 terminals are believed to mainly modulate ongoing cellular activity (Chung et al. 2002; Petrof and Sherman 2013).

According to the above criteria, our data can be viewed as an indication that Po synapses effectively “drive” with higher efficacy M1wk neurons, while playing a more “modulatory” role on S1BF neurons. In fact, modulatory-like features of the postsynaptic potentials evoked by Po synapses in S1BF neurons have been observed with intracellular recording methods (Viaene et al. 2011a). A recent “in vitro” study (Mo and Sherman 2019) examined the currents produced by Po synapses in motor cortex neurons, and found relatively large excitatory postsynaptic currents, which showed paired-pulse depression to the optogenetic activation of Po terminals. However, the study did not address the receptor mechanisms involved.

## Concluding remarks

Hierarchically wired architectures may be the cellular substrate for the predictive and error-detection forebrain computations that produce skilled motion and active sensing (Larkum 2013; Ahissar and Assa 2016). In the cerebral cortex, layer-specific patterns of arborization (Crick and Koch 1998; Markov et al. 2014), synaptic bouton size and specific receptor mechanisms (Sherman and Guillery 2013) have all been related to different hierarchical levels of information processing.

In this context, the present study demonstrates that Po signals can simultaneously reach the motor and sensory cortical modules that are involved in active sensing with the vibrissae. Temporal coherence in Po thalamocortical inputs might thus control functional connectivity between the separate cortical areas, which involved in the complex cognitive task that is active whisker exploration in rodents (Saalmann et al. 2012; Nakajima and Halassa 2017; Schmitt et al. 2017). Moreover, our finding that the Po axon terminals target specific layers, have markedly different bouton sizes and involve different receptor mechanisms raises the intriguing possibility that Po signals may have different hierarchical range/operational value in M1wk than in S1BF.

## Electronic supplementary material

Below is the link to the electronic supplementary material.

Supplementary material 1 (DOCX 14 KB)

Supplementary material 2 (TIF 9365 KB)
