# Supplementary material for: Posterior thalamic nucleus axon terminals have different structure and functional impact in the motor and somatosensory vibrissal cortices
Source: Brain Struct Funct. 2019 Mar 27;224(4):1627–45. doi: 10.1007/s00429-019-01862-4 (PMC6509070; doi:10.1007/s00429-019-01862-4)
Supplement: Supplementary file 1 — Supplementary material 1 (DOCX 14 KB) [file 429_2019_1862_MOESM1_ESM.docx]

**Supplementary Materials**

**Supplementary Materials Table SM1**

|  | **% Axon length per layer** | | | **% Varicosities per layer** | | |
| --- | --- | --- | --- | --- | --- | --- |
| **Layer** | **S1BF** | **M1wk** | ***p*** | **S1BF** | **M1wk** | ***p*** |
| **1** | 12.8 ± 3.2 | 7.6 ± 3.3 | 1 | 11.8 ± 1.4 | 9.8 ± 3.5 | 1 |
| **2-4** | 14.9 ± 5.7 | 47.6 ± 7.7 | 0.004 | 12.5 ± 6.4 | 55.5 ± 8.1 | 0.000 |
| **5a** | 48 ± 7.7 | 23.3 ± 7.2 | 0.006 | 56.2 ± 11.2 | 18.4 ± 7.1 | 0.000 |
| **5b** | 11.3 ± 1 | 14.2 ± 3.8 | 1 | 11.6 ± 4.2 | 8.7 ± 2.5 | 1 |
| **6** | 12.8 ± 3.2 | 7.3 ± 9.5 | 1 | 7.9 ± 1.6 | 7.5 ± 5.2 | 1 |
| ***2-3** | 7.7 ± 3.8 | - | - | 7.4 ± 5.1 | - | - |
| ***4** | 7.2 ± 1.9 | - | - | 5.1 ± 1.4 | - | - |

**Table SM1. Stereological estimations of the axonal length and varicosities percentages per cortical layer in S1BF and M1wk areas (mean ± SD)**

Stereological estimations of axonal length and varicosity numbers per layer in S1BF and M1wk. Measurement were obtained from whole are samplings in three different BDA labeling experiments for each area. P-values are indicated. * In the case of S1BF, estimations were also performed for layers 2-3 and 4 separately.

**Supplementary Materials Figure SM1**

Length density and density of varicosities in S1BF. Stereological estimates of axonal length density and varicosities density per individual cortical layer of area S1BF in each of the 3 cases included in the study
