# Supplementary figures and images for: Posterior thalamic nucleus axon terminals have different structure and functional impact in the motor and somatosensory vibrissal cortices
Source: Brain Struct Funct. 2019 Mar 27;224(4):1627–45. doi: 10.1007/s00429-019-01862-4 (PMC6509070; doi:10.1007/s00429-019-01862-4)

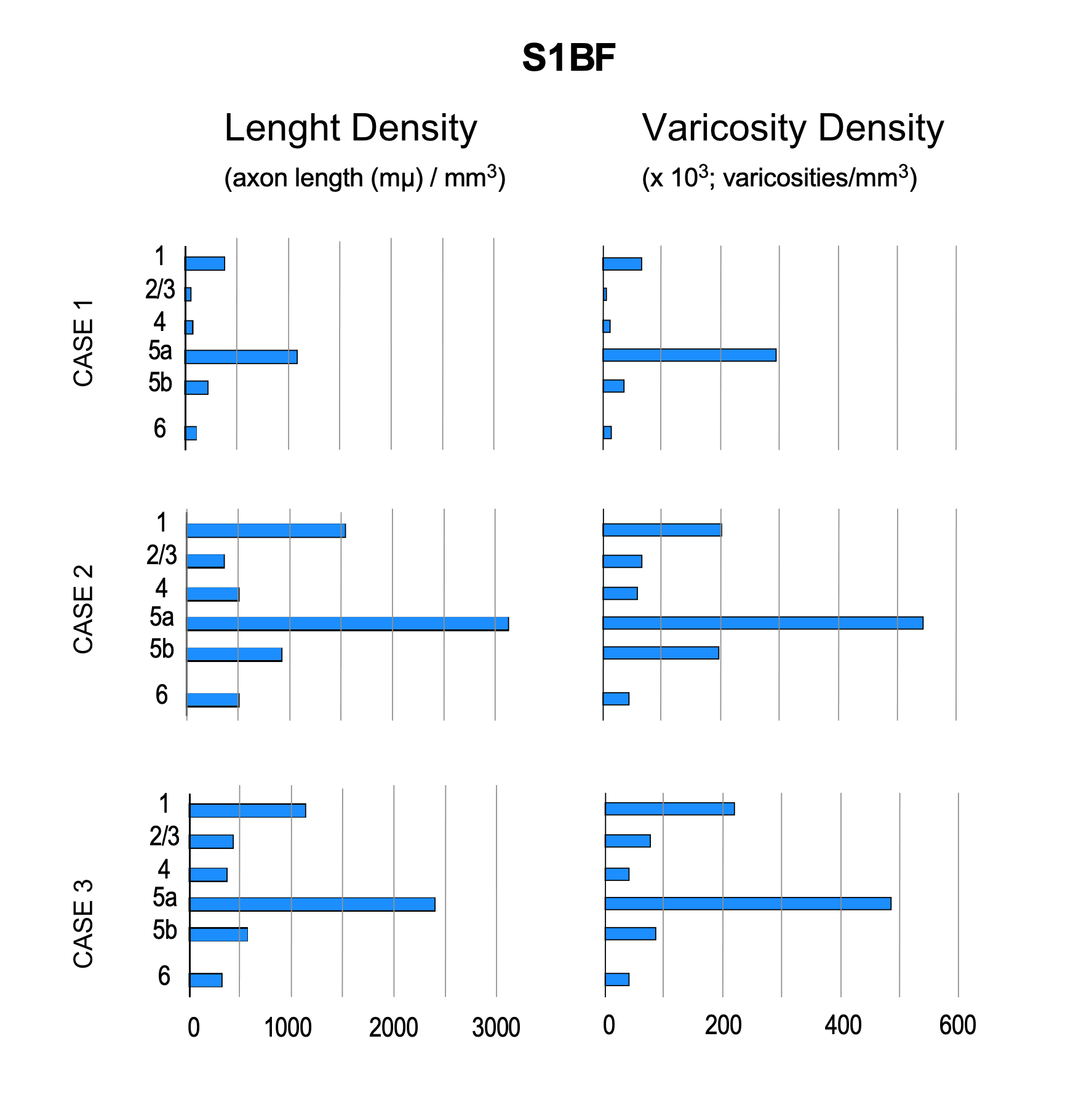

Supplement: Supplementary file 2 — Supplementary material 2 (TIF 9365 KB) [file 429_2019_1862_MOESM2_ESM.tif]
